# Blood Urea in Nephritis

**Published:** 1915-11

**Authors:** 


**Blood Urea in Nephritis.** Albert J. Underhill of Baltimore, N. Y. Med. Jour., Sept. 25, reports several cases, including acute nephritis with suppression, due to mercuric chlorid. Widal and Javal place the normal amount of urea in the blood at 15-50 centigrams per liter. Marshall and Davis have shown that injection of urea in dogs is followed by increase in the urine, the excess being eliminated within 24-36 hours. The author makes a full report of examinations on a case of bichlorid poisoning (5 grams dissolved in beer). There was anuria for two days, the blood urea on the day after ingestion being 84 c.g. per liter. It gradually rose to 5.32 grams per liter, on the 12th day, when the urine urea was 10.99 grams in 700 c.c. of urine. On the 26th day, the blood urea was 48 c.g., the urine urea 41.49 in 2500 c.c. On the 29th day when the patient was discharged, the blood urea was only 27 c.g. per liter. (Note: The absolute amount of urea in the blood may be estimated at about 5 or 6 times the amount per liter). In another bichlorid case, the blood urea rose to 4.83 grams per liter on the 14th day of observation, as much as 4.2 grams having been obtained from the stools for 12 hours. In another, the maximum was 5.66 per liter on the 15th day after ingestion, when the patient died. In idiopathic cases of nephritis, the amounts of urea in the blood are usually much lower, for instance, 80 c.g. and up to 3 grams but in a fatal case of uraemia, 4.65 grams was noted 3 days before death. (Note: It is interesting to observe that these investigations partially substantiate the early, literal sense of the term uraemia, though urea itself is only slightly toxic).

